# Physical Activity and Cognitive Impairment in a Group of Adults with Down Syndrome from North-Eastern Romania

**DOI:** 10.3390/jcm13195829

**Published:** 2024-09-29

**Authors:** Nicoleta Lefter, Irina Mihaela Abdulan, Alexandra Maștaleru, Maria-Magdalena Leon, Cristina Rusu

**Affiliations:** 1 Faculty of Medicine, “Grigore T. Popa” University of Medicine and Pharmacy, 700115 Iasi, Romania; lefter.nico@gmail.com; 2 Department of Medical Specialties I, “Grigore T. Popa” University of Medicine and Pharmacy, 700115 Iasi, Romania; alexandra.mastaleru@gmail.com (A.M.); leon_mariamagdalena@yahoo.com (M.-M.L.); 3 Clinical Rehabilitation Hospital, 700661 Iasi, Romania; 4 Department of Mother and Child Medicine, “Grigore T. Popa” University of Medicine and Pharmacy, 700115 Iasi, Romania; abcrusu@gmail.com

**Keywords:** down syndrome, physical activity, cognitive decline

## Abstract

**Background**: Down syndrome (DS) is the most prevalent chromosomal condition, with the average life expectancy significantly rising from 25 years in 1983 to 60 years in 2020. People with DS generally can participate in most physical activities that individuals without the disability can, despite some physical limitations. However, the varying degrees of cognitive deficits can present challenges when it comes to performing physical activities. **Methods**: We conducted a prospective, cross-sectional study in the Cardiovascular Rehabilitation Clinic from the Clinical Rehabilitation Hospital from Iași, Romania, between July 2022 and February 2024 that included 28 patients diagnosed with DS. We collected data regarding age and comorbidities and performed several tests (MMSE, timed-up-and-go test, and handgrip strength). **Results**: The group consisted of 11 (39%) females and 17 (61%) males with a mean age of 28.07 ± 9.51 years. The weight profile of the study group differed according to the degree of physical activity: 62.50% of those with moderate physical activity were moderately overweight, while 75% of those with minimal activity were overweight or obese. The muscle strength and MMSE score was higher in the group of patients who performed moderate physical activity. Regarding comorbidities, there was a statistically significant difference in the percentage of patients with hearing loss among those with minimal physical activity. **Conclusions**: Our results provide an update on the very limited data available. The study indicates that people with DS are generally less physically active and face unique health challenges (hearing loss, intellectual disabilities, and osteoarticular pathologies).

## 1. Introduction

Down syndrome (DS) is the most prevalent chromosomal condition, with the average life expectancy significantly rising from 25 years (1983) to 60 years (2020). Adults with DS are particularly susceptible to several pathologies due to their chromosomal abnormalities. The most prevalent condition is early-onset Alzheimer’s disease (AD), significantly affecting cognitive function and quality of life. Additionally, individuals with DS are at higher risk for heart conditions such as congenital heart defects and autoimmune disorders like hypothyroidism. They also experience a higher incidence of sensory impairments, such as hearing and vision problems [1].

Intellectual disability and AD in adults with DS represent distinct but often overlapping conditions. Intellectual disability is a lifelong condition characterized by below-average cognitive functioning and limitations in adaptive skills, present from birth. In contrast, AD is an age-related neurodegenerative disorder that can develop later in life, leading to progressive cognitive decline and memory loss. While individuals with DS have an inherent intellectual disability, the onset of AD introduces additional cognitive impairments and behavioral changes beyond their baseline condition. Distinguishing between these two requires careful assessment, as symptoms of Alzheimer’s can be mistakenly attributed to the pre-existing intellectual disability [2].

The main challenge is the limited amount of research available, highlighting the necessity for further research to back up guideline suggestions for nearly every issue. For children with DS, the high estimated prevalence of comorbidities (such as congenital heart defects, thyroid disease, and obstructive sleep apnea) warrants routine screening, even for asymptomatic individuals. When it comes to adults with DS, there is insufficient understanding of the prevalence and severity of comorbid health conditions and their respective risk factors. The current efficacy, risks, and benefits of screening asymptomatic adults with DS for the same conditions recommended for those without this disease are being questioned. Future research is needed for each guideline topic. For the first time, the GLOBAL Workgroup utilized existing evidence from a systematic literature review and employed a standardized and transparent methodology to develop clinical practice recommendations for adults with DS. Even so, the recommendation’s status appearing in this guide is ‘weak’ in most cases [3]. Moreover, in Romania, there are no such guidelines.

**Figure 1:**
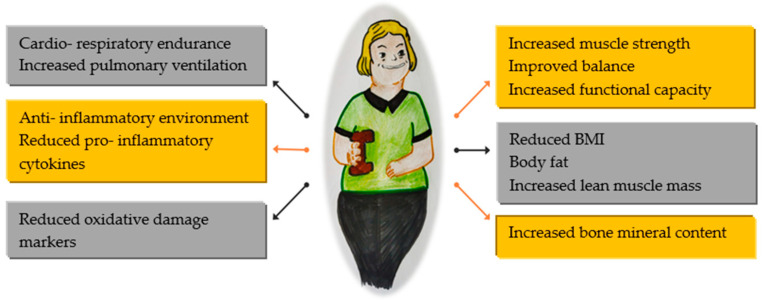
Benefits of physical activity in global health.

Regular physical activity can improve physical function, especially in individuals with reduced ability (Figure 1). However, it is important to note that consistent exercise may not always prevent functional limitations associated with aging, disability, or illness.

People with DS are recommended to engage in the same amount of physical activity as those without disabilities. The WHO Physical Activity Guidelines suggest at least 150 min of moderate-intensity aerobic activity per week if possible. Moderate and vigorous intensities can be combined for physical activities. These activities should be spread out during the day and done for at least 10 min each to maximize the benefits. Health improvements are directly related to the amount of physical activity performed. If someone is unable to complete the recommended exercise safely, they should engage in activities that they can tolerate. However, it is crucial to consult a physician before starting any new physical activity [4].

All these recommendations should be tailored to the needs of these patients as they face various health challenges that can impact their ability to participate in physical activities. These include cardiovascular issues such as congenital heart defects, which may lead to heart failure and symptoms like shortness of breath and fatigue. Obstructive sleep apnea can also reduce energy levels and make exercise more tiring. Visual and hearing impairments can affect balance and coordination, making certain activities more difficult. Additionally, hypothyroidism, which is common in this population, can cause fatigue, weight gain, and muscle weakness, further hindering their ability to exercise. Furthermore, cognitive impairments can make it difficult for them to learn new skills and participate in activities that require quick decision making, such as team sports.

Cognitive impairment in adults with DS is a well-documented aspect of the condition, characterized by a range of intellectual and developmental challenges. Individuals with DS typically exhibit varying degrees of cognitive deficits, including difficulties with memory, language comprehension, and problem-solving skills that could become a challenge in performing physical activities [4]. In addition, treating medical conditions or a major mental health disorder is usually welcomed by adults with DS and their family members. However, medical professionals who are not familiar with caring for adults with DS may find it challenging to effectively provide such treatments.

Adults with DS in Romania frequently encounter considerable financial difficulties, as numerous families find it challenging to cover the costs of essential medical care and supportive services that could improve their quality of life. Nonetheless, allocating resources for prevention and proper care, such as physical activity programs and cognitive therapies, can greatly contribute to the well-being and cognitive abilities of individuals with DS, ultimately reducing long-term healthcare expenses and boosting their autonomy.

The purposes of this research are to contribute to the clinical understanding of these complex individuals by highlighting the characteristics of the adult population with DS in north-eastern Romania and to find a possible link between physical activity and cognitive status.

## 2. Materials and Methods

### 2.1. Type of Study

We conducted a cross-sectional study at the Cardiovascular Rehabilitation Clinic of the Clinical Rehabilitation Hospital (Iași, Romania) from July 2022 to February 2024. The study involved 28 patients who were diagnosed with DS, previously confirmed through genetic testing.

### 2.2. Patients Selection

In the specified timeframe, we found 28 patients with DS who met the inclusion criteria out of 505 patients admitted to our clinic. The inclusion criteria were an age over 18, genetic testing that confirms the diagnosis, and signing of the informed consent. Patients with DS who had recent surgery, neoplasia, conditions associated with increased risk of falling, refusal/failure to perform the proposed tests, or severe cognitive impairment due to other factors were excluded from the study. Specifically, individuals diagnosed with AD or severe neurodegenerative disorders, severe mental health disorders like major depressive disorder or schizophrenia, recent traumatic brain injuries, or severe uncorrected sensory impairments were also excluded due to their potential impact on cognitive function related to DS.

### 2.3. Data Collection

A single investigator performed history, tests and physical examinations and collected general information (age, sex, and comorbidities).

The determination of socio-economic factors included information on standard of living, marital status, involvement in social activities, and education.

The Mini Mental State Examination (MMSE) is a powerful tool for evaluating mental status. With 30 questions covering five areas of cognitive function, it provides a comprehensive assessment. A perfect score is 30, and a score of 24 or lower indicates cognitive impairment (sever impairment—under 10 points; moderate—10–19 points; mild—20–24 points). The MMSE is efficient, taking only 10–15 min to administer, making it suitable for regular and repeated use [5].

The measurement of muscle force was conducted using an electronic dynamometer, labeled as EH101. During the test, the participant was seated on a standard chair with the shoulder abducted and the elbow positioned near the trunk and flexed at a 90° angle, while the wrist was in a neutral position (thumb facing upwards). The opposite arm remained relaxed underneath the thigh. Handgrip strength was determined by having participants perform one trial to familiarize themselves with the procedure and one measurement trial using both the dominant and non-dominant hands. The maximal contraction was measured over a 4 s duration with verbal encouragement. While some guidelines and studies offer specific cut points and percentiles for muscle weakness, it is anticipated that clinically significant ethnic-, sex-, and age-specific categories for muscle weakness based on handgrip strength (HGS) measurements, similar to BMI thresholds, will be developed in the future. In our research, we utilized the cutoff points provided by The European Working Group on Sarcopenia in Older People (EWGSOP, the Sarcopenia Working Group), which are less than 30 kg for men and less than 20 kg for women [6].

The timed-up-and-go test (TU & Go) is the most used test worldwide to measures the dynamic balance and functional mobility in adults, elderly, as well as in the neurological population [4,6]. The TU & Go involves a patient standing up from a chair, walking three meters, turning, returning, and sitting back down. The test measures the total time taken, and the resulting score in seconds is correlated with the risk of falls [7]. The TU & Go is commonly used in many studies as a cutoff point for mobility limitations, typically between 10 and 14 s [5]. This test is highly correlated with balance, gait speed, and functional capacity, and it has been proven to be a reliable and valid measurement of independent ambulation while also reflecting age-related decline [8,9].

### 2.4. Ethics Committee

All participants in the study provided informed consent prior to enrollment. This study received approval from the Ethics Committee of both the “Grigore T. Popa” University of Medicine and Pharmacy Iași (208/9 July 2022) and the Iași Clinical Rehabilitation Hospital (13/22 June 2023).

### 2.5. Statistical Analysis

We conducted statistical analysis using SPSS 20.0 (Statistical Package for the Social Sciences, Chicago, IL, USA). Categorical variables were expressed as percentages and compared using the chi-square test. The Shapiro–Wilk test was used to assess the normality of distribution for continuous variables. Variables with a normal distribution are presented as mean values with standard deviations and were compared using the Student’s *t*-test. Non-normally distributed continuous variables are presented as medians with interquartile ranges and were compared using the Mann–Whitney U test. The threshold for statistical significance was set at a value of *p* ≤ 0.05 for all analyses.

## 3. Results

The group consisted of 11 (39%) females and 17 (61%) males. Age ranged from 20 to 55 years with a mean of 28.07 ± 9.51. All patients were unmarried, without a partner but with family support, and they were all non-smokers.

In examining demographic factors related to physical activity, we found that adults who engaged in moderate physical activity tended to be older, had higher levels of education, came from two-parent families in 75% of cases, and had fewer medications as part of their chronic prescription (Table 1).

Not all patients received treatment for chronic conditions. Therefore, it is important to also acknowledge the approach taken in managing the associated pathologies:

- Nonbarbiturate hypnotics to manage insomnia in three out of five patients;
- Proton pump inhibitors to treat chronic gastritis in 7 out of 17 patients;
- Thyroid hormone replacement therapy for 7 out of 24 patients;
- Analgesics/NSAIDs for lumbago in 2 out of 11 patients;
- Preventative treatment for thrombophilia using antiplatelet antiaggregant in one patient;
- Psychiatric intervention with SSRI antidepressants/tricyclics to address behavioral disorders in four out of eight patients;
- Anticonvulsants for one patient with a confirmed diagnosis of epilepsy.

The weight profile of the study group differed according to the degree of physical activity: 62.50% of those with moderate physical activity were moderately overweight, while 75% of those with minimal physical activity were overweight or obese.

The muscle strength was higher in the group of patients who performed moderate physical activity, both in the dominant (statistically insignificant) and non-dominant hand (*p* = 0.002). In addition, within this group, the duration for executing the TU & Go was reduced. (Table 2).

MMSE values ranged from 10 to 23, with a mean of 14.79 ± 3.96. Despite not being statistically significant, we observed a higher mean MMSE in patients who performed moderate physical activity (Table 3).

The MMSE components did not reveal statistically significant differences between patients with minimal versus moderate physical activity, but several aspects are worth mentioning; orientation, memory, language, and execution had higher mean values in patients with moderate physical activity, with memory being better in those who performed their activity exclusively at home (Table 4).

When it comes to related conditions, every patient had a confirmed level of intellectual disability. A noticeable difference was found in the number of patients experiencing hearing loss among those with limited physical activity (Table 5).

One-third of the patients had behavioral issues. The initial examination of the medical history showed that there are significant impacts on social interactions, emotional well-being, and daily functioning. Symptoms such as changes in appetite, sleep problems, irritability, withdrawal from social activities, and loss of interest in activities that were once enjoyed could point to underlying anxiety and depression and were grouped under these conditions. Furthermore, impulsive behaviors and difficulty in understanding boundaries necessitate ongoing support in social settings. It is important to note that behavioral issues can be exacerbated by sleep problems and overstimulation in hospital environments.

**Figure 2:**
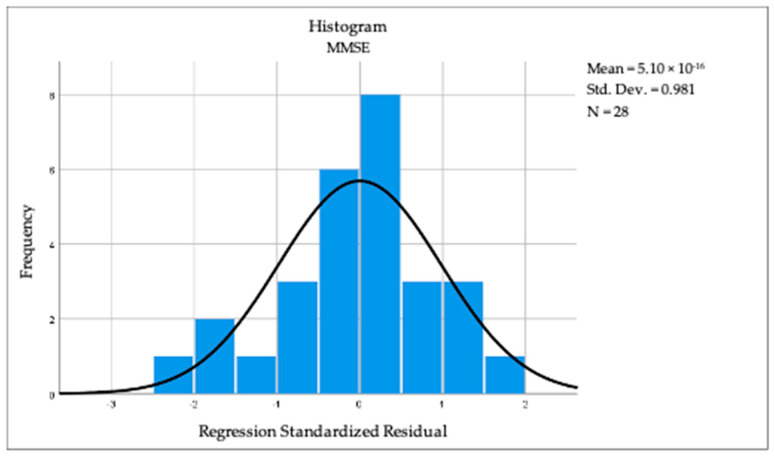
Histogram revealing the linear regression between cognition and dominant hand muscular strength.

**Figure 3:**
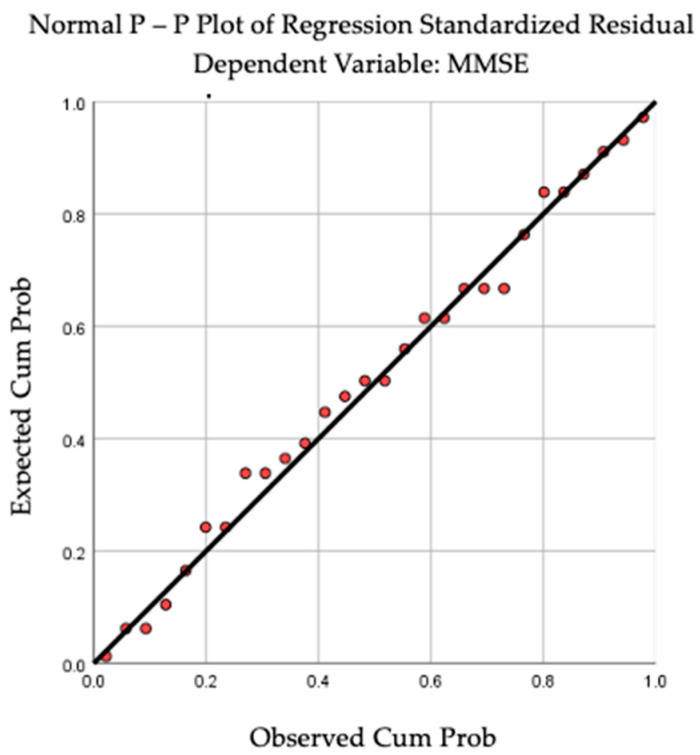
P-P Plot revealing the linear regression between cognition and dominant-hand muscular strength.

By applying linear regression analysis, we observed a statistically significant correlation between cognition and the dominant-hand muscle force (R = 0.410, R square = 0.168, *p* = 0.030). The above-mentioned results can be observed in Figure 2 and Figure 3. For BMI (R = 0.040, *p* = 0.840) and TU & Go (R = 0.297, *p* = 0.125), no statistical significance was observed in the linear regression analysis.

## 4. Discussion

In recent decades, advancements in medical treatment have increased the life expectancy of people with DS. Consequently, they now need more extensive healthcare services in the family practice setting. The primary care provider should strive to integrate and coordinate specialized care to address the patient’s long-term needs.

Most published studies include pediatric population. Considering the limited data on adult patients and the associated pathologies, designing guidelines for the management of these patients has proven to be a continuous challenge [10].

Even though theoretically, they can benefit from medical services to the same extent as a patient without genetic disorders, there are certain challenges regarding the practical approach:

- Individuals with DS are more likely to experience behavioral and psychiatric conditions such as autism spectrum disorders, ADHD, or anxiety [11]. In both children and adults, exposure to a new environment, the stress of collecting blood tests, or clinical examination itself limits visits to the doctor to only emergencies;
- Limited access to care is due to socioeconomic factors, low availability of specialized healthcare providers, and inadequate healthcare infrastructure.

In the north-eastern part of Romania, there is a single genetics center. Due to the lack of extensive clinical guidelines and the limited experience of doctors in different medical fields, patients in this region are typically directed to the genetics center for further guidance. When caring for adults with DS, clinicians frequently face the challenge of determining when to adhere to the standard guidelines for adults without DS.

Providing appropriate care for individuals with DS can result in substantial cost savings for healthcare services. For example, research in South Carolina found that adults with intellectual disabilities had more than 21,000 preventable visits to the emergency department, resulting in over USD 35 million in healthcare expenses due to situations that could have been managed by the GPs [12].

### 4.1. General Characteristics

In our study, we found that over half of the participants were from single-parent households, but all of them had support and resided with their families. It was observed that the primary caretaker in these families was the mother.

Regarding education, the vast majority attended classes in schools for children with special needs. Even so, half of them graduated eight classes, and 10% had not been in school at all.

Several studies indicated that individuals with DS typically require more time to learn new skills. It may be necessary to break down new skills into smaller steps for them and provide additional repetition to help them retain what they have learned [13].

It is important to remember that the gap in skills and learning between children with DS and other children tends to widen as they get older. By the time they reach secondary school, this gap can become quite significant. It is important to recognize that people with DS keep learning and developing new skills as they move into their teenage and adult years. They have the potential to make progress and learn throughout their lives, just like anyone else. A student with DS is more likely to thrive in a school that embraces and supports inclusion as part of its culture and where the diverse learning needs of all students are recognized and addressed. Therefore, limiting access to education and hindering its progress can be detrimental to these individuals.

### 4.2. Physical Activity

The consistently low levels of physical activity among adults with DS, as seen in our study and the literature, may be partially attributed to biological factors. It is possible that decreased muscle tone and autonomic dysregulation make it harder for individuals with DS to meet the recommended activity levels compared to the general population.

Studies have found that individuals with DS exhibit reduced peripheral oxygen uptake and altered autonomic function during exercise. This can result in a reduced response to physical activity, including a lower heart pulse and impaired regulation of blood flow to the extremities [14].

Agiovlasitis et al. [15] found that people with DS have lower step-rate thresholds for moderate-to-vigorous physical activity and vigorous activity compared to those without DS. Furthermore, adults with DS have a higher metabolic equivalent workload at a given step rate than adults without this condition. This may explain why adults with DS tend to engage in lower levels of physical activity compared to the general population.

Fewer steps are needed for adults with DS to reach the recommended MVPA threshold compared to the general population.

The same research mentions that individuals with DS who are between 140 and 170 cm in height can achieve the moderate-intensity physical activity recommendation by accumulating 2280 to 3030 steps in 30 min, five days a week. They can also accomplish this target in shorter sessions of at least 10 min each. Likewise, meeting the vigorous intensity recommendation involves accumulating between 1890 and 2040 steps in 15 min, five days a week. Combining moderate- and vigorous-intensity activities is also advantageous.

The handgrip test is validated for evaluating the strength of upper limb muscles in adults with intellectual disabilities. It has shown excellent reliability with a score of 0.94 [16]. Terblanche and Boer utilized it to assess physical fitness in adults with DS [17], and Boer and Moss confirmed its excellent reliability index of 0.98 [18].

In our study, we measured the handgrip strength using a dynamometer in both the dominant and non-dominant hands. We found that muscle strength was lower in both hands for all individuals. However, we observed that individuals who performed moderate physical activity had a better handgrip strength. Research has indicated that people with DS have reduced muscular strength in both their upper and lower body compared to the general population. This weakness in the lower extremities significantly affects their capacity to carry out everyday tasks like walking and climbing stairs [19].

In 2019, Coelho-Junior et al. [20] discovered that individuals with DS who are adults exhibited higher levels of body fat, reduced bone mineral density, reduced lean mass, and physical performance comparable to or lower than that of older adults with sarcopenia.

The observation related to muscle strength and sarcopenia can also be made in our DS group, in which the average handgrip strength was clearly lower than the limits for sarcopenia [6].

The reasons stated above might clarify why people with DS tend to have diminished cardiovascular fitness, higher obesity rates, and decreased muscular strength in comparison to the general population [21].

The TU & Go was verified for frail older adults [22]. In individuals with DS, Terblanche and Boer applied the test as well [17], and its consistency was later assessed by Boer and Moss, showing an outstanding reliability index of 0.94 [18]. In our study, the test was performed in a lesser amount of time by those who were engaged in moderate physical activity. Studies have demonstrated that adults with DS exhibit reduced physical fitness and activity levels compared to their typically developing peers. They also tend to engage in more sedentary behavior, regardless of age [23].

### 4.3. Cognitive Impairment

Dealing with individuals with DS presents unique challenges due to behavioral disorders and changes in mental status. Identifying coexisting mental illnesses can be particularly difficult, so understanding common psychiatric disorders can be beneficial if a medical evaluation does not provide a diagnosis. Research suggests that around 25% to 30% of adults with DS have psychiatric disorders [24]. Common findings include dementia, depression, conduct disorders, and obsessive-compulsive disorder, while schizophrenia, alcoholism, and substance abuse are less common.

Our patients had similar rates of behavioral disorders to previously presented data, and all of them had a medically confirmed degree of intellectual disability.

The cognitive development of individuals with DS is often limited, particularly in the early stages. Expressive language skills tend to lag behind receptive language skills. In Romania, there is a lack of specific studies on adults with DS, so psychologists rely on standardized tests such as the Raven Progressive Matrices, the Wechsler Adult Intelligence Scale (WAIS), and the Mini-Mental State Examination (MMSE). The Romanian legislation recognizes the MMSE as a valid assessment for individuals with disabilities, despite its limitations [25].

There are different tests available to assess cognitive abilities in children and adults. The Colored Progressive Matrices Test (MPC) is useful for measuring nonverbal intelligence in children, individuals with mental retardation, and the elderly. The Wechsler and Binet–Simon tests mainly focus on verbal abilities. The Binet–Simon tests are designed to assess intellectual abilities in children aged 3 to 13, with tasks that increase in complexity as children grow older [26]. The WAIS, on the other hand, measures intelligence and cognitive ability in older adults and adolescents. It introduced a non-verbal performance scale to address the criticism that the Binet scale was too focused on verbal skills [27,28].

These tests have overlapping components with the MMSE, especially in terms of measuring numeracy skills, literacy, and general cognitive developments through tasks such as sentence repetition, naming familiar objects, and copying figures are used to assess language and memory skills. More complex tasks like performing sequences and demonstrating comprehension and logical thinking are also part of the assessment.

Many researchers have recognized the necessity of adapting cognitive assessment tools to specific populations, including those with developmental disorders. Other researchers used brief comprehensive measures of neuropsychological functioning derived from mental status examinations that were designed to assess dementia in individuals without intellectual disabilities [29,30]. Although the criteria used to confirm cognitive decline are not absolute, it is very important to recognize any evidence of gradual loss of function other than the normal effects of aging [31]. This refers to alterations in memory and other cognitive functions, orientation, emotional control, motivation, and social behavior [32]. Some neuropsychological tests originally developed to diagnose dementia for the general population, like MMSE, have been modified for patients with intellectual disabilities [33].

The Modified Mini Mental Status Examination-Down Syndrome (MMMSE-DS) used by Krinsky-McHale et al. [29] included testing orientation to person, place, and time, along with the ability to name objects, recite the alphabet, count forwards and backwards, print letters and numbers, draw simple geometric patterns, memorize by repetition, and fine motor praxis. The total score on the MMMSE-DS is 74.

Even if the MMSE adaptations were effective and show some degree of sensitivity, some failed to reach statistical significance in longitudinal data analysis [34].

At the end of the 20th century, an EEG study included MMSE to screen out subjects with DS who did not fulfill the criteria for dementia [33]. However, other authors have suggested that the MMSE does not appear to be a useful tool because subjects with DS without dementia score less than 24, which is the diagnostic cut-off point for the general population, and a “floor effect” has even been identified.

There are few studies using the original MMSE on subjects with DS. In 2008, M. Boada et al. [35] analyzed the role and usefulness of the Severe Impairment Battery (SIB) and the MMSE in the cognitive assessment of aging subjects with DS. The SIB and the MMSE were administered to 45 subjects with DS (16 with AD and 29 without dementia), and the DMR questionnaire was given to their caregivers. The researchers applied the original MMSE with a maximum score of 30, with any score below 24 indicating cognitive deterioration in the general population. This study found a statistically significant correlation between the two objective cognitive tests, the MMSE and SIB, and the subjective DMR cognitive subscale (r = −0.495; *p* = 0.001) (r = −0.507; *p* = 0.0005). This finding suggests that the original MMSE may be of use to assess cognitive function in adults with DS. From the clinical standpoint, the need to monitor cognitive function makes it advisable to use objective scales such as the MMSE and the SIB. The study concluded that, while the SIB and MMSE may not be relevant to the diagnosis of Alzheimer dementia in subjects with DS, they may play a highly useful role when it comes to monitoring cognitive deterioration and dementia in these subjects. The authors advocated for their widespread routine use in order to reduce examination time, allowing comparison with mainstream populations.

Future attempts to adapt the MMSE should also take into account educational status and cultural backgrounds. Many of these adults have limited formal education, which affects their ability to perform tasks involving reading, writing, and math. In Romania, the situation is exacerbated by the limited engagement of adults with DS in medical care, which impedes research efforts and reduces the incentive for hospitals to invest in specialized diagnostic tools.

In our research, we chose to use the MMSE despite its acknowledged limitations within this specific population. Our decision was influenced by practical considerations. We acknowledge that the MMSE is not fully validated for adults with DS, and its use in this context should be approached with caution. In Romania, despite the substantial population of individuals with DS, recruiting a sufficient number of participants for such validation poses an exceptional challenge.

Our findings showed that there was no ‘floor effect’, as the scores varied widely and were well distributed across different levels of performance. The majority of the participants were in school, and 57% even graduated from high school.

We propose several recommendations for the future adaptation of the MMSE. For instance, questions regarding temporal and spatial orientation could be simplified by asking for only essential information such as the year and month or the city and street, avoiding more complex details. In assessing immediate memory, it is recommended to use simple and easily memorable words, while in the case of attention and mental calculation, successive subtractions might be replaced with more accessible tasks, such as counting backward from a smaller number or repeating short sequences of digits. Finally, for assessing short-term memory, providing a simple context to facilitate recall is advisable.

Our findings align to some extent with those reported in existing research on cognitive function in adults with DS. To advance the understanding of cognitive decline in this population, it is imperative to replicate this study with a larger sample size and implement annual follow-ups.

This study represents the foundational stage of our work, and we are optimistic about the feasibility and potential impact of this approach. We are determined to continue our efforts regarding the development of an adapted MMSE for the DS population in Romania. However, overcoming obstacles like social stigma and educational constraints, along with promoting greater cooperation with relevant authorities as well as establishing strong partnerships among individuals with DS, their families, caregivers, clinicians, and researchers, is essential to achieving our objectives.

### 4.4. Comorbidities

When assessing cognitive impairment, we need to consider acquired cognitive deficits such, as dementia which is more likely to develop over time. Current studies have demonstrated the benefits of physical activity in halting the advancement of AD and cognitive decline in this high-risk group [36]. Despite the possible advantages of physical activity for people with DS, prior research has shown that these patients engage in less physical activity than the general population [37]. Many of these studies, however, included only children [38].

Considering the higher occurrence of obesity in individuals with DS and the heightened cardiometabolic risks linked to weight gain, it is crucial to have a deeper understanding of their physical activity habits [39].

Our research found that two-thirds of the patients were overweight or obese. Among those who did minimal physical activity, 75% were overweight or obese, while 62% of those who engaged in physical activity outside the home had a normal weight range.

Aside from obesity, patients with DS experience various related conditions that impact their daily physical activity.

It is common for both adults and children with DS to experience hearing impairment. This high rate can be attributed to various factors such as a weakened immune system, structural differences in the face and ear, and an increased likelihood of chronic ear disease. A study involving 51 adults with DS revealed that it is important to always consider hearing impairment as a potential cause or contributing factor when individuals exhibit behavior changes, poor mental health, and/or a decline in cognitive abilities [40].

Various elements that can lead to vestibular dysfunction involve low muscle tone, flexible joints, reduced reflexes, slower reaction time, and problems with balance. The vestibular system works together with vision and proprioception to support the maintenance of balance and motor coordination [41].

In our research, over 50% of the patients experienced some level of hearing loss. We noted a notable statistical difference depending on their level of physical activity. Among those who participated in minimal indoor activity, 70% experienced this problem, while only 12.5% of those who engaged in outdoor activities had the same issue.

Sleeping disruption is another frequent problem that can have negative effects on mental and physical health. Individuals with learning disability are at particularly high risk. Most studies on sleep disturbances in this population have focused on children and relied on information from parents, which does not always align with objective sleep monitoring results. There is limited information on the frequency, severity, and health impacts of sleep disorders in adults despite the fact that individuals with learning disabilities live longer [42]. The negative effects are on both mental and physical health. Individuals with a learning disability are at a particularly high risk. However, in our study, only 17.85% of our patients suffered from sleep disorders, with no significant difference regarding the physical activity. One possible explanation for the low number is the underdiagnosis of this pathology despite its multiple implications. We acknowledge the profound impact of insomnia on overall health, and we believe that is essential to engage the expertise of a psychiatrist to address this issue effectively.

In 2021, a study by Gimenez et al. [43] highlighted the adverse effects of untreated sleep disorders on mental and physical status, behavior, and cognitive function. Furthermore, a separate research study involving 47 non-demented adults with DS found that increased levels of sleep disturbance, as measured by actigraphy, were associated with poorer episodic memory, executive function, motor planning, and coordination [44]. The study monitored sleep patterns over seven nights using a wrist-mounted actigraphy accelerometer. It relied on input from the adult with DS and a caregiver through a sleep diary.

The results of a 2019 study showed strong associations between sleep issues, body mass index, excessive daytime sleepiness, and various health and psychological problems in adults with DS, as reported by 100 family caregivers in an online survey. The most reported issues included lack of daytime energy (45%), excessive daytime sleepiness (38%), irritability (33%), difficulty staying awake during the day (30%), falling asleep during the day (26%), taking daytime naps (25%), feeling drowsy (25%), and feeling miserable during the day (21%). These findings highlight the difficulty of maintaining a regular exercise routine for individuals with a sleep disorder [45].

It is worth mentioning that in this study, the occurrence and types of comorbidities differed in some aspects compared to previous research [46]. The occurrence of a single case of epilepsy among the participants may be attributed to their relatively young age, as epilepsy is more prevalent in older individuals.

We encountered no cases of diagnosed sleep apnea; however, due to atypical symptoms and the absence of prior polysomnographic assessments, definitive exclusion remains uncertain.

The absence of specific diagnoses such as autism, ADHD, schizophrenia, and bipolar disorder from our study can be attributed to regular health check-ups only being mandatory until the age of 18. Additionally, expertise in these disorders was limited in Romania during the participants’ childhood years.

The diagnosis of behavioral impairment would need further investigation to identify an underlying cause, and this would require a thorough psychiatric consultation by a psychiatrist.

For example, even to this day, there is a lack of official data on autism, leading to underdiagnoses of autism spectrum disorders. The cost of therapy per patient averages between 1500–2000 euros per month, often unfeasible for the majority of families.

### 4.5. The Burden of the Neuropsychiatric Symptoms

Psychiatric disorders impact 20–30% of individuals with DS [47], These conditions often manifest through behaviors like stubbornness, obsessiveness, hyperactivity, and conduct disturbances.

Behavioral challenges can have a significant impact on the self-sufficiency of adults with DS. These challenges can make it harder for them to handle daily tasks, maintain employment, and participate in social interactions. Issues such as impulsivity, anxiety, and difficulty regulating emotions can make it challenging for individuals to stick to schedules, follow directions, and interact appropriately with others. These are crucial skills for independent living. Moreover, these behavioral challenges may lead to a greater need for supervision and support, which limits opportunities for autonomy. Therefore, it is essential to address behavioral issues through targeted interventions to improve the independence and overall quality of life for adults with DS.

It is essential to have a specialized physician assess neuropsychiatric symptoms using tools like the Neuropsychiatric Inventory Questionnaire-Severity Scale (NPIQ) [48]. Additionally, Lawton’s Activities of Daily Living (ADL) and Instrumental Activities of Daily Living (IADL) scales can help evaluate their impact on daily life.

### 4.6. Future Steps in Monitoring Physical Activity

Family participation is crucial for establishing a consistent workout routine. Improving the fitness and strength of individuals with DS can be achieved through exercises such as walking, jogging, and cycling on a stationary bike, which can help in enhancing their respiratory and cardiovascular systems. Additionally, muscle-strengthening exercises can contribute to enhancing balance, overall strength, and muscle fitness. These activities, however, can become challenging, causing stress through repetition, environment, and existing behavioral disorders (emotional infantilism). They can be replaced with pleasant, simple activities that also involve a reward. As a result, everyday tasks like walking to class and carrying books or groceries can become easier as their strength and endurance increase. This improvement in physical abilities can lead to boosted self-esteem and confidence, contributing to an overall better quality of life.

Health and fitness professionals can assess whether individuals with DS meet these targets using accelerometers or affordable pedometers. It is important to note that pedometers may be less accurate for individuals with DS compared to those without DS, with piezoelectric pedometers displaying better precision in DS population [49].

One potential future approach for monitoring physical activity involves using smart devices such as watches to transmit real-time information about physical activity (such as step count, calories burned, heart rate, and sleep quality) to the medical team through an application. This would involve additional expenses for families, who would need to purchase the smartwatches, as well as for the medical system, which would need to implement protocols and special informational systems.

Better understanding of the activity and inactivity patterns can assist healthcare professionals in offering more effective guidance to adult patients with DS regarding managing their energy levels and making lifestyle changes to prevent and address obesity and other metabolic disorders.

With recent research highlighting the potential advantages of physical activity in reducing the risk of dementia in individuals with DS [36], advising on physical activity for this at-risk group may also provide further benefits. Adults with DS may benefit non-medically from physical activity as well. These benefits could include a chance for social engagement and enjoyment, a sense of accomplishment, and an improvement in day-to-day tasks [50,51].

The limitation of this research refers to the challenges of studying physical activity in adults with DS. The small population size results in limited sample sizes, reducing the generalizability of findings. Within this study, there is significant variability in cognitive and physical abilities, and this does now allow to draw broad conclusions. One decisive factor consists of environmental and social factors like family support and community resources.

In our study, physical activity was self-reported. By effectively monitoring physical activity levels using the devices mentioned above, we could motivate and assist individuals with DS in staying physically active. This support could potentially help reduce health issues experienced by this population.

Another aspect that is worth mentioning is the fact that the individuals who were part of our research did not come back for follow-up appointments (regardless of our research), even though we advised them to do so. We believe this is because of several reasons, such as the high-stress atmosphere of the hospital and financial limitations. The hospital environment can be very daunting and cause anxiety, and financial challenges make it difficult for them to receive regular medical care. Consequently, there are chances for continuous care and assistance that are being missed.

Based on the challenges we faced during this study and the feedback from patients and caregivers, we developed some recommendations that we believe highlight the main pillars in the care of adults with DS (Table 6).

## 5. Conclusions

Adults with DS often face unique health challenges, and when compounded by hearing impairment, these challenges can significantly impact their levels of physical activity. This study indicates that adults with DS are generally less physically active than their peers without disabilities. Several factors contribute to this reduced activity, including physical, cognitive, and social barriers.

This study shows that predisposition to obesity can make physical activity more strenuous. When hearing impairment is also present, these physical challenges are exacerbated. Hearing loss can impede communication and social interactions, making it harder for individuals to engage in group activities or follow instructions in physical settings. Also, the association of intellectual disability has been proven to make it particularly challenging to understand and respond to auditory cues or safety warnings during exercise.

Including regular physical activity into the lives of individuals with DS can lead to significant improvements in mental status and overall quality of life. It supports cognitive function, emotional well-being, behavioral regulation, social skills, and physical health. Encouraging and facilitating access to physical activity should be a key component of care strategies for individuals with DS.

## Figures and Tables

**Table 1:** General characteristics of the study group.

	Total Group (N = 28)	Minimal Activity (N = 20)	Moderate Activity (N = 8)	*p*-Value
Age, years (mean ± SD)	28.07 ± 9.51	27.55 ± 8.43	29.37 ± 12.38	0.65
Education, N (%) - No classes - Primary - Gymnasium - High school	3 (10.71%) 9 (32.14%) 6 (21.42%) 10 (35.71%)	2 (10%) 8 (40%) 4 (20%) 6 (30%)	0 (0%) 2 (25%) 2 (25%) 4 (50%)	- 0.47 0.78 0.33
Family support	28 (100%)	11 (100%)	17 (100%)	-
Single-parent family	17 (61%)	14 (70%)	3 (37.50%)	0.12
Unemployed	28 (100%)	20 (100%)	8 (100%)	-
Medication, (mean ± SD)	1.07 ± 1.21	1.25 ± 1.20	0.65 ± 1.06	0.22

**Table 2:** Assessment of weight status and physical activity.

	Patients Enrolled (N = 28)	Minimal Activity (N = 20)	Moderate Activity (N = 8)	*p*-Value
BMI, kg/m^2^, (mean ± SD)	30.17 ± 8.89	30.20 ± 6.45	30.09 ± 13.86	0.97
Weight status, N (%) - Normal range - Overweight - Obesity class I - Obesity class II - Obesity class III	10 (35.71%) 7 (25%%) 4 (14.28%) 4 (14.28%) 3 (10.71%)	5 (25%) 6 (30%) 4 (20%) 3 (15%) 2 (10%)	5 (62.50%) 1 (12.50%) 0 (0%) 1 (12.50%) 1 (12.50%)	0.05 0.35 - 0.87 0.85
Muscle strength, kg (mean ± SD) - Dominant hand - Non-dominant hand	14.51 ± 3.11 14.32 ± 3.90	13.03 ± 3.21 13 ± 3.33	16.25 ± 2.17 17.63 ± 3.33	0.61 0.002
Timed-up-and-go test (s)	12.96 ± 3.87	13.75 ± 3.94	11 ± 3.07	0.08

BMI—body mass index (kg/m^2^). Normal range—18.5–24.9; overweight 25.0–29.9; obesity class I—30.0–34.9; obesity class II—35.0–39.9; obesity class III—over 40.

**Table 3:** Assessment of cognitive impairment.

	Patients Enrolled (N = 28)	Minimal Activity (N = 20)	Moderate Activity (N = 8)	*p*-Value
MMSE, (mean ± SD)	14.79 ± 3.96	14.40 ± 4.18	15.75 ± 3.41	0.42
Cognitive impairment - Mild - Moderate	22 14.23 ± 3.52	22 13.56 ± 3.45	- 15.75 ± 3.41	- 0.14

**Table 4:** MMSE components and physical activity.

MMSE	Patients Enrolled (N = 28)	Minimal Activity (N = 20)	Moderate Activity (N = 8)	*p*-Value
Orientation	4.25 ± 1.60	4.20 ± 1.67	4.38 ± 1.50	0.79
Attention	0.89 ± 0.83	0.8 ± 0.83	1.12 ± 0.83	0.36
Memory	3.71 ± 1.01	3.85 ± 1.03	3.38 ± 0.91	0.27
Language	2.28 ± 0.53	2.20 ± 0.52	2.50 ± 0.53	0.18
Execution	3.60 ± 1.28	3.35 ± 1.38	4.25 ± 0.70	0.94

**Table 5:** Comorbidities and physical activity.

Comorbidities	Total Group (N-28)	Minimal Activity (N = 20)	Moderate Activity (N = 8)	*p*-Value
Disk hernia, N (%)	3 (10.71%)	3 (15%)	0 (0%)	-
Lumbago, N (%)	11 (39.28%)	9 (45%)	2 (25%)	0.34
Hearing loss, N (%)	15 (53.57%)	14 (70%)	1 (12.5%)	0.004
Behavioral issues N (%)	10 (35.71%)	7 (35%)	3 (37.5%)	0.90
Intellectual disability, N (%)	28 (100%)	20 (100%)	8 (100%)	-
Oftalmological issues, N (%) - Strabismus - Myopia - Astigmatism	3 (10.71%) 10 (35.71%) 5 (17.85%)	3 (15%) 6 (30%) 3 (15%)	0 (0%) 4 (50%) 2 (25%)	- 0.33 0.54
Insomnia, N (%)	5 (17.85%)	4 (20%)	1 (12.5%)	0.65
Obesity, N (%)	11 (39.28%)	9 (45%)	2 (25%)	0.34
Chronic venous insufficiency, N (%)	12 (42.85%)	7 (35%)	5 (62.5%)	0.13
Urinary continence, N (%)	16 (57.14%)	11 (55%)	5 (62.5%)	0.72

**Table 6:** Possible strategies in the management of adults with DS.

Regular Physical Activity	Encourage structured exercise programs tailored to the individual’s abilities, as regular physical activity can improve cardiovascular health, enhance cognitive function, reduce anxiety, and promote overall well-being.
Comprehensive Health Monitoring	Implement routine health assessments, including regular screenings for thyroid function, cardiovascular health, and early signs of AD, to detect and manage conditions that could impact cognitive function.
Personalized Behavioral Interventions	Provide access to personalized behavioral therapies that address specific challenges such as anxiety, depression, and social difficulties, helping to improve social interactions, emotional well-being, and daily functioning.
Nutritional Support	Collaborate with nutrition specialists to ensure a balanced diet that supports cognitive health and physical well-being. This involves identifying and addressing any nutritional deficiencies and promoting positive and healthy eating patterns.
Family and Caregiver Education	Educate families and caregivers on best practices for supporting their cognitive and emotional needs, including strategies for creating a supportive home environment and understanding the importance of routine and structure.
Cognitive Stimulation	Encourage participation in cognitive activities, such as puzzles, memory games, and educational programs, to stimulate mental function and slow cognitive decline.
Access to Specialized Care	Mental health support, physical therapy, and occupational therapy.
Social Engagement Opportunities	Facilitate social interaction through community programs and specially designed social groups, which can help reduce isolation and improve overall quality of life.
Stress Reduction	Create supportive environments that minimize stress, particularly in healthcare settings, to reduce anxiety and improve the willingness of individuals to engage in regular medical follow-ups.
Long-Term Care Planning	Encourage early planning for long-term care needs, including financial planning and decisions regarding living arrangements, to ensure a stable and supportive future.

## Data Availability

The data that support the findings of this study are available from the corresponding author upon reasonable request.
